# Emotional Deprivation in Children: Growth Faltering and *Reversible* Hypopituitarism

**DOI:** 10.3389/fendo.2020.596144

**Published:** 2020-10-07

**Authors:** Alan David Rogol

**Affiliations:** Department of Pediatrics, University of Virginia, Charlottesville, VA, United States

**Keywords:** emotional deprivation syndrome, growth, hypopituitarism, growth hormone, stress

## Abstract

Emotional deprivation can lead to growth faltering of infants and children. The mechanism(s) involved differ in that for infants, the major metabolic problem is inadequate energy intake for growth. In young children, it is likely that the emotional deprivation causes a syndrome not only of growth faltering, but with bizarre behaviors, especially with regard to food: hoarding, gorging and vomiting, hyperphagia, drinking from the toilet, and eating from garbage pails. Other disturbed behaviors include, poor sleep, night wanderings, and pain agnosia. The pathophysiology appears to be reversible hypopituitarism, at least for the growth hormone and hypothalamic-pituitary- adrenal axes. The review begins with an historical perspective concerning stress, children and growth and then moves to the issue of hospitalism, where young infants failed to thrive (and died) due to inadequate stimulation and energy intake. Refeeding programs at the end of World Wars I and II noted that some children did not thrive despite an adequate energy intake. It appeared that in addition taking care of their emotional needs permitted super-physiologic (catch-up) growth. Next came the first notions from clinical investigation that hypopituitarism might be the mechanism of growth faltering. Studies that address this mechanism from a number of observational and clinical research studies are reviewed in depth to show that the hypopituitarism was relieved upon removal from the deprivational environment and occurred much too quickly to be due to adequate energy alone. These findings are then compared to those from malnourished children and adoptees from emerging countries, especially those from orphanages where their psychosocial needs were unmet despite adequate caloric intake. Together, these various conditions define one aspect of the field of psychoneuroendocrinology.

## Introduction

Growth refers to the increase in body size over time. Within the broad range of normal for age, sex, and ethnicity, size attained is an accepted sign of good general health for children. Growth has two major components, amplitude (size attained) and *tempo*, the rate at which the parameters change; both depend on many factors, including the socioeconomic conditions into which a child is born and reared (1). Malnutrition, specifically energy deficit, if severe, is quickly followed by slowing of growth [stunting; height-for-age Z-score <-2 (HAZ) using the World Health Organization (WHO) standards]; of note, deficits in weight often appear before those in length or height (2).

The first 1,000 days of life from conception to the second birthday are a critical period when good nutrition and appropriate care are essential. Malnutrition is associated with a double-burden: stunting of linear growth and subsequent obesity and non-communicable diseases in adults (2). A reduction in rate of growth occurs first, whether associated with malnutrition or with chronic illness. With increasing severity and duration, it may become permanent as stunted adult height. Nevertheless, catch-up in adult height can occur even with longstanding starvation as noted in Norwegian children following the German occupation during World War (WW) II (3). Here, catch-up growth (CUG) is characterized by the height velocity above the upper limit of normal for age and sex at least one year following a transient period of growth retardation (4)

Growth faltering, a growth rate below which is inappropriate for an infant’s or child’s age and sex is often associated with delayed neurobehavioral development during infancy and early childhood, and both are assumed to have common etiologies. Rapid early post-natal growth, including that of the brain, requires adequate nutrition; early brain development also requires environmental stimulation. A systematic review of nutritional (and other) interventions on length- or height-for-age and developmental outcomes (5) noted significant positive effects of re-feeding on growth and cognitive, language, motor, and socio-emotional scores. Moreover, the effect sizes for growth parameters were significantly associated with the effect sizes for the cognitive and motor scores.

The variability in growth among individuals is considerable as many normally growing and developing infants cross weight-for-age percentiles in the first 6 months of life. In one study, for example, 32% of infants moved up or down two major weight percentiles, 39% two major height percentile, and fully 62% two major percentiles of weight-for-height (6). This likely represents genetic variation in the expression of growth potential with the removal of the constraints of the intrauterine environment.

### Energy Requirements for Growth

Re-feeding of malnourished infants and young children assumes an “adequate” diet (and micronutrients) that will replace the energy deficit and permit normal growth and perhaps catch-up growth, characterized by a height velocity above normal limits for at least for a year after growth inhibition (4).

Does replenishment of missing calories (total energy deficit) in stunted children completely explain growth patterns noted during re-feeding? In some, perhaps a majority of infants and children, growth whether normal or even catch-up in length and weight is highly correlated. In a re-feeding study, the length and weight of 51 underweight-for-age infants were followed over a 9-month interval (7). All gained weight; however, 20/51 started catch-up growth after 4 months of the added nutrition, while 28/51 experienced CUG in length as evident in increased height (Ht)-SDS after 9 months of nutritional rehabilitation. Those who showed CUG as weight-for-height did not change the BMI SDS noting balanced growth. Infants who gained more weight (average gain >7 g/day) showed significant increases in weight-for-height, BMI-SDS, and Ht-SDS compared to their status before the nutritional rehabilitation. It was suggested that weight gain greater than physiologic (~6.5 g/day for similarly aged children) supported the energy requirement for CUG. Among 28 children who increased their BMI following increased energy intake, daily weight gain was considerably greater than those who did not, 8.6 ± 5.8 g/day *versus* 3.3 ± 2.2 g/day; estimated height velocity the two groups was, respectively, 7.4 ± 3.6 cm/y and 5.7 ± 2.8 cm/y. The latter was interpreted as indicating that energy required for linear growth was not stored, but was used in the processes that permitted the increased height velocity. Although there may be some questions about the quality of the diet used in any re-feeding trial, especially the essential and conditionally essential amino acid content (8), there are compelling data defining outliers to the concept that underweight, even stunted, malnourished children require only enough energy with proper macro- and micro-nutrients to begin to grow and even display CUG.

In a related study, 16 infants, 3–24 months of age, were referred for the evaluation of growth failure; all had normal birth weight and length (9). The infants gained weight with initial hospitalization with “minimal” or “high intensity” mothering, but required adequate calories. At home (and with a member of the experimental team present to guarantee the infant was provided with adequate energy intake), the infants continued to gain weight. Some of the mothers noted increased intake from what the child had taken previously, while others admitted to the withholding of food for a variety of reasons. These observations lead the authors to conclude that the non-organic failure-to-thrive of such infants was due to inadequate energy intake (9), consistent with earlier data which suggested that older infants actually required the energy intake of younger, rapidly growing infants (10).

### Other Factors

Nutrition and inflammation are inextricably intertwined in the life of malnourished infants with inputs from enteric infection, diet, and maternal “factors”. Higher degrees of inflammation as measured by markers such as C-reactive protein were associated with lower levels of IGF-1 among children in developing areas of the world (11). It should be noted, however, that inflammation itself is associated with underlying pathogen carriage, which is also associated with poor nutritional status (12) and shorter stature (13). Nevertheless, these processes are complex and in many cases there is overlap of suppression of IGF-1 from underlying inflammation, nutritional insufficiency, and growth hormone resistance. The three interact at the growth plate where rate of chondrogenesis dictates linear growth through regulatory inputs of circulating hormones, paracrine and intracrine factors, and an array of cytokines, nutrients and other regulatory molecules [reviewed in (14)].

Emotionally deprived infants, especially younger ones, are malnourished as are those who have been raised in some institutions and orphanages. Nevertheless, some emotionally deprived older infants and children are growth stunted, but not obviously malnourished, i.e., weight-for-height and BMI Z-scores are within the normal range (2, 15).

## Historical Perspective

### After World War I

Growth in weight and height of German school-age children with long-standing energy deficits during re-feeding following previous starvation during World War I has been recently reported (16, 17). Based on original observations of earlier studies of large group re-feeding of children after the war—whether raised in intact families of all social strata (18, 19), orphaned by the war, or from underprivileged families (20), the authors noted that children from underprivileged families had growth outcomes very different from the former two, *despite* adequate caloric replenishment. Children from underprivileged families continued to show sub-normal growth in height and weight gain, whereas the vast majority of children in the former two groups showed rapid catch-up growth, reaching 3 to 5 cm within 4 to 6 weeks (17). For example, in a nominal 8 week program German children were brought to Switzerland (Schweizerfürsorge, Swiss charity program) to live with foster families or in inns and lodges. They were given an adequate diet and were permitted to play and interact as children. They showed significant gains in weight and height. Although the exact height velocity was difficult to quantify because some children stayed longer than the originally scheduled time, the vast majority of children showed substantial gains (**Table 1**). A marked increase in hemoglobin concentration, and salutary alterations in temperament were also noted [Abderhalden, 1920] (18).

**Table 1:** Height and weight changes upon refeeding of German children over approximately 6 to 8 weeks in Switzerland after WW I.

Height and Weight Changes upon refeeding*						
			Boys		Girls	
Age	Weight change	Height change	BMI**	Height**	BMI**	Height**
(years)	(kg)	(cm)	SDS	SDS	SDS	SDS
(8–14)						
mean	3.31	2.94	-1.05	-1.06	-1.10	-1.20
SD	1.5	2.46				

*Data from Hermanussen, et al. (16).

**World Health Organization (WHO) Standard.

In a related study, 30 girls from Germany in another Swiss community were also evaluated [Bloch, 1920] (19). With re-feeding and the new living conditions, the average height increase was approximately 3 cm in 6 weeks and a much greater increase in the tuberculin response was noted; the latter indicated that the suppressed immune system was returning to the physiological state with re-feeding. In contrast to the preceding, results for a group of 512 children referred for very serious undernutrition to a pediatric hospital in Berlin suggested different results (20). The children were 6 to 14 years of age. The weights and heights of the children were very much lower at the initial observation (**Table 2**) compared to the studies described above. The hospitalized children gained 3–4 cm in 6 months, a rate much lower than described by Bloch [1920] (19) in a span of six weeks. The gain in weight, although smaller than in the other studies, was not compensated by a robust gain in height, i.e., the BMI Z-score increased.

**Table 2:** BMI and height of children orphaned during WW I before refeeding at a pediatric hospital in Berlin.

BMI and height of children orphaned during WW I before refeeding^1^				
	Boys		Girls	
Age	BMI	height	BMI	height
years	SDS^2^	SDS^2^	SDS^2^	SDS^2^
6.5	-0.12	-2.22	-0.11	-2.64
7.5	-0.21	-2.84	-0.14	-2.07
8.5	-0.84	-1.89	-0.89	-2.04
9.5	-0.50	-2.05	-0.70	-2.38
10.5	-0.87	-2.36	-1.00	-2.41
11.5	-1.12	-2.33	-1.98	-2.03
12.5	-1.29	-2.42	-1.31	-2.69
13.5	-1.68	-2.46	-1.34	-1.99
14.5	-2.05	-2.85	-1.74	-2.70

^1^Data from Hermanussen et al. (16).

^2^Based on World Health Organization (WHO) standards.

Original data noted in Hermanussen, et al. (16).

It should be noted that backgrounds of the children evaluated in the preceding studies varied considerably. The children studied by Abderhalden (18) and Bloch (19) came from intact social backgrounds irrespective of social class. They had a smaller height deficit at the beginning of the re-feeding program and demonstrated rapid catch-up growth. In contrast, the large group of children studied by Goldstein (20) consisted mainly of malnourished and orphaned children and children of underprivileged families. The sample was much shorter at the start of the re-feeding period and had mean heights between -2.0 and -2.8 SDS (relative to the current WHO standard). More than half of the children were 5 to 15 cm below the average for age, or equivalent to 1 to 3 years of growth behind. Although they experienced significant weight gain, CUG in height was comparatively less after 26 weeks.

Results for the latter sample (20) show parallels to patterns observed in children of *contemporary* middle- and low-income countries among whom it was suggested that providing food to low-income, stunted [height-for-age, HAZ<-2SD] populations may be beneficial for some, but “…may be detrimental for others” and may contribute to obesity, and by inference, lower height velocity compared to weight gain (21). These may impact healthspan, the period of one’s life when free of serious illness (healthy), as well as lifespan (22). As noted earlier, stunting is a common indicator of childhood undernutrition (2).

### Failure to Thrive in Young Infants and Hospitalism

Data from the early 20^th^ century were followed by observations of infant behavior, growth (and mortality) in institutions where young infants were domiciled for custodial care for extended periods of time, also noted as “hospitalism”, or the non-organic failure of infants to thrive in institutions due to emotional deprivation (23, 24). Previous studies focused on young infants and were generally centered on maternal factors, and emotional reactions of young infants in response to sensory stimuli were addressed (24). Subsequently, it was noted that the energy intake necessary for accelerated growth was similar to that of rapidly growing infants younger than one year in spite of the apparent inefficiency in energy metabolism; i.e., excessive relative to actual body weight (9, 10). Nevertheless, it was possible to distinguish those <2–3 years as also experiencing *maternal deprivation*. In contrast, some data suggested a potentially different mechanism in children >2 or 3 years, specifically *reversible* hypopituitarism (considered below) (25, 26).

The linear growth and early behavioral development of young children in low and middle income countries was recently evaluated in a meta-analysis (27). The pooled adjusted standardized mean difference in cross-sectional cognitive ability per unit increase in height-for-age Z-scores (HAZ) for children ≤2 years was +0.24 (95% CI, 0.14–0.33) and +0.09 for children >2 years (95% CI, 0.05–0.12). Prospectively, each unit increase in HAZ for children >2 years old was associated with a +0.22 SD increase in cognition at 5 to 11 years after multivariate adjustment (95% CI, 0.17–0.27). The HAZ was also significantly associated with earlier age at walking and more advanced motor development scores (P < 0.05). Overall, the observational evidence suggests a strongly positive association between linear growth during the first 2 years of life and cognitive and motor development. Given the trends in the relationships, it was suggested that effective integration of environmental, educational, and stimulation interventions for infants can produce a large positive effect on linear growth (27).

The overwhelming preponderance of data suggests that factors other than caloric replacement (energy) *may* affect a child’s general response to re-feeding following nutritional stunting, even in the absence of overt gastrointestinal malabsorption. Some children will show remarkable catch-up in linear growth and weight as noted in the two post-WW I studies (18, 19), although others will not as in studies of war orphans (20) and specific patients (25, 26, 28), especially when the children remained in adverse environments. The concepts described above were based on observations in the late 19^th^ and early-to-mid 20^th^ centuries, mainly in Germany and Austria. Failure-to-thrive and mortality were strikingly high in infants who were hospitalized in foundling hospitals or were ill enough to be in regular hospitals. However, the concept that psychosocial conditions *may* affect early growth can be dated to Emperor Frederick II (Emperor of the Holy Roman Empire and King of Italy and Germany) in the 13^th^ century as noted by his chronicler, the monk Salimbene di Adam [in Haskins, (29)]. Frederick II was well ahead of his time in seeking the results of actual experiments. In one such linguistic “experiment” chronicled by GG Coulton on the “vile bodies of hapless infants” (29, 30),

…“bidding foster mothers and nurses to suckle and bathe and was the children, but in no wise to prattle or speak with them; for he would have learnt whether they would speak the Hebrew language, which had been first; or Greek, or Latin, or Arabic, or perchance the language of their parents of whom they had been born. But he labored in vain for the children could not live without clapping of the hands, and gestures and gladness of countenance, and blandishments” [GG Coulton “St. Francis to Dante”; London: David Nutt, 1906 pp 242–243.] (30)

Otherwise, unexplained growth failure in association with socially stressful conditions and significant CUG when a child’s caregiving environment improve were noted as early as 1828 with the story of Kasper Hauser [as related in (31)]. In the late 19^th^ century, two “case studies” were presented to the Section of Diseases of Children at the Annual Meeting of the British Medical Association held in Edinburgh, July, 1898 (32). The patient vignettes described children from a foundling asylum who had persistent decreases in weight and activity associated with progressive “exhaustion”. The children had all of the signs of marasmus and upon evaluation lost weight and nitrogen daily. Autopsies showed atrophic areas of the intestinal tract, but also areas of normal bowel histology which were not consistent with severe bacterial infection or toxic substances. The latter were considered unlikely as suggested by experiences with such infants who had no evidence of transmission between children as would be expected with common gastrointestinal or respiratory infections. Baginsky concluded that the children succumbed to faulty nutrition, nursing or general care, for *“want of sufficient and proper care”*, the latter in its fullest sense [Baginsky, 1899, p. 1084] (32).

Discussions among pediatricians and psychiatrists considered the need to keep the surroundings clean to prevent infection (small, individual rooms, masked, hooded, and scrubbed nurses and physicians moving cautiously to prevent stirring up the air or dust with the resident bacteria *versus* usual care as we know it today). Parents were not permitted to visit and mothers were masked and gowned when nursing their infants. Most of these infants did not thrive. The literature noted loneliness (33) and anaclitic depression: an emotional deficiency disease of infancy which included withdrawal, apathy, *weight loss*, sleep disturbance, and decreased development quotient merged with hospitalization without intervention (23, 34). Observations in a foundling hospital suggested that very few of the children were of normal height or weight, and that the mortality rate was much higher than average, in spite of cleanliness and adequate food, In contrast, observations in a prison hospital where mothers fed and interacted with their infants, and where toys, perceptual stimuli and opportunities for movement were available, the infants thrived and the mortality rate was considerably lower (23).

“A progressive development of emotional interchange with the mother provides the child with perceptive experiences of its environment. The child learns to grasp by nursing at the mother’s breast and by combining the emotional satisfaction of that experience of its environment. He learns to distinguish animate objects from inanimate ones by the spectacle provided by the mother’s face in situations fraught with emotional satisfaction. The interchange between mother and child is loaded with emotional factors and it is in this interchange that the child learns to play. He becomes acquainted with his surroundings through the mother carrying him around; through her help he learns security in locomotion as well as in every other aspect. This security is reinforced by her being at his beck and call.” [Spitz, 1945; p. 59] (23)

Physicians, developmental psychologists, and psychoanalysts provided different explanations on understanding hospitalized babies as a contented baby, who had all his or her needs met and stood a good chance of survival; as a baby who lagged behind in development through inheritance and lack of mental and physical stimulation and thus purportedly posed a threat to the hereditary make-up of the population; or as a baby who had not experienced love and interest and had thus fallen behind in social behavior (35). In a summary of many of the earlier studies, the following were noted about institutionalized infants, who may show (36):

- Emotional, physical, and intellectual deficits
  - Perhaps due to deprivation of “maternal love”
  - This occurs only after specific affective responsiveness has been achieved around the age of six months
- Perceptual deprivation is also important—the absolute or relative absence of tactile, vestibular, and other forms of stimulation
  - These are important for proper language development and *can be provided* within an institutional setting

## Maternal Deprivation Syndrome/Psychosocial Short Stature

The link between psychosocial wellbeing and linear growth was presaged by Fried and Mayer (37) based on observations of children in an institution that cared for dependent and neglected children:

…“socio-emotional adjustment plays not merely an important, but actually a crucial role among all the factors that determine individual health and physical growth and development” and

“…socio-emotional disturbance tends to affect physical growth adversely and that growth failure so caused is much more frequent and more extensive that is generally recognized even at an institution with proper care, physical and psychological”. [Fried and Mayer, 1948, p. 455]

Four remarkable growth curves were presented illustrating diminished height velocity in association with accentuated psychological maladaptation (37).

Others have presented similar series of patients, for example, chronically undernourished children who failed to thrive despite the absence of demonstrable disease (38). The author, a social worker, noted that the children often appeared pale and scrawny with failure to gain weight and to attain developmental milestones. Although the etiology of this condition was not clear, weight gain in some children within the hospital environment or during foster care outside of the original home was noted, leading to the speculation that the social environment may be a powerful influence on the course of an infant failing to thrive, i.e., anticipating the maternal deprivation syndrome (38).

Subsequently, the characteristics of 9 children, 4 to 16 years, who fulfilled the strict criteria for psychological short stature were described (39). The children showed remarkable linear growth and changes in personality structure upon removal from the adverse environment. No endocrine data were reported, but the children fulfilled the “final” criterion of accelerated growth and salutary changes in behavior following removal from the original domicile (39).

The characteristics of 10 patients who experienced parental rejection as the important factor for their clinical presentation were also described (40). All presented with short stature, subnormal weight-for-height, and an enlarged abdomen, but experienced very rapid reversal of all physical signs and linear growth acceleration upon removal from an adverse environment *without* therapy other than normal hospital care. The authors suggested that the degree of malnutrition was insufficient to explain the subnormal height velocity associated with parental rejection.

In a subsequent study of 185 of children with failure-to-thrive, 50% were considered to have experienced “environmental deprivation” based on a thorough medical and psychosocial history (41). Of interest, routine (non-endocrine) laboratory investigation *rarely* was helpful in delineating a proper diagnosis without a specific indication from the history or clinical evaluation. Later in the 1940’s children and adolescents presented with moderate-to-severe growth failure, often with a bizarre personal history that may have included signs and symptoms related to behavior or body structure and growth status (42, 43). An abnormal and disturbed relationship between the primary care giver and the child, most often the child’s mother, was also noted. Others have reported elements of the Munchausen syndrome by proxy as important in some children diagnosed with a deprivation syndrome (44, 45):

- parent or other caregiver fabricates an illness
- the child has multiple encounters for medical assessment, often resulting in multiple procedures
- parent denies the cause of the child’s illness
- acute symptoms and signs of the illness stop when the child and perpetrator are separated

The behavioral signs of psychosocial short stature include most or all of the following, although some may overlap with those noted above:

- a history of unusual eating and drinking behavior, reversible on change of domicile, such as eating from a garbage can and drinking from a toilet bowl, stealing food, alleged picky eating and rejecting food at the table, polydipsia and polyphagia, possibly alternating with gorging and vomiting and with self-starvation
- history of such behavioral symptoms as enuresis, encopresis, social apathy or Inertia, defiant aggressiveness, sudden tantrums, crying spasms, insomnia, eccentric sleeping and waking schedule with “prowling” at night, pain agnosia, and self-Injury, all occurring *only* in the growth-retarding environment
- delayed motor development with improvement on removal of the child from the domicile of abuse
- delayed Intellectual growth, reversible on change of domicile by as much as 30 to 50 IQ points
- a history of pathologic family relationships, Including unusual cruelty and neglect, either somatic or psychic or both (45).

These signs and symptoms are quite variable and the most severely affected children will have multiple indications of high severity. In addition, children with psychosocial short stature differ significantly from those with stunting due primarily to nutritional inadequacy in terms of irritability, apathy, decreased social responsiveness, and anxiety and attention deficits.

Auxologic observations of children with psychological short stature include significant short stature and diminished height velocity (25). The short stature following normal birth weight, length and infantile growth is most often proportional and skeletal maturation (bone age) is delayed; the latter is more often better correlated to the height age than to chronological age (25). Occasionally, growth faltering will begin in the teenage years (46, 47).

## Emotional Deprivation as Psychosocial Short Stature

The condition presently called psychosocial short stature (PSS) is the focus of the remainder of this report to illuminate the concept that the psyche might influence physical growth and how this might occur. PSS was also known as psychosocial dwarfism, deprivation short stature or deprivation dwarfism; however, the term “dwarfism” is presently considered pejorative (48). Etiologic mechanisms include nutritional deficit per se; increased systemic inflammation mainly from infectious agents (11), and various endocrine hypotheses. There may be multiple and interrelated mechanisms depending on the age of the child: PSS type I in infants <2 years (10, 49, 50) and type II for those >2 to 3 years (25, 43). However, it has been noted that the specific symptom of hyperphagia, mainly in concert with polydipsia and hoarding and scavenging for food, will distinguish two subtypes of type II PSS, which may differ by etiology (51–54). It is this dietary sign that may predict reversibility of GH deficiency (51). One may also consider those in the type II category as spanning a spectrum of psychosocial deprivation. At one pole would be the patients described by Powell and co-workers (25) with many of the behavior signs noted above and denoted “hyperphagic” by Skuse and co-authors (51). These children are GH deficient during the time that they reside at the adverse environment and experience CUG upon removal. At the other pole would be those with fewer behavioral signs and apparently less growth faltering, but who do not have GH deficiency when tested and who may or may not have a growth spurt with removal from the domicile. The latter must be tempered by the caveat that some may have recovered GH sufficiency when tested some days later in the hospital environment. The GH deficient phenotype is closely associated with hyperphagia, polydipsia, and food-seeking behaviors, but with a normal BMI (52). The latter was interpreted as sign that at least in this sub-group of children with non-organic failure to thrive, that chronic undernutrition was not the prominent pathophysiologic mechanism (55). The latter was complemented with observations that stunted children world-wide are often difficult to re-feed and do not show CUG; that children in the study group did not eat within the hospital setting for more than a few days and did not increase the BMI at the initial follow-up; and that GH responses (basal and in response to stimulation) in those with protein-calorie malnutrition were robust at entry and decreased upon re-feeding (56, 57).

Although there are more experimental data for the hypopituitarism hypothesis, the data are not exclusive as there are at least significant influences of nutrition, energy availability and micronutrients, and inflammatory system dysregulation. Energy availability *alone* may not be sufficient to support normal or catch-up growth after extended periods of inadequate nutritional energy intake, as noted in the post-WW I re-feeding experiments described above and the detailed analysis of the studies that began in the 1940’s and recounted below. The diagnosis of psychosocial short stature as defined here is *confirmed* when a child grows normally or has CUG, and shows behavioral and developmental improvements upon removal from the deleterious environment—without specific medical, hormonal, or psychological treatment. Are there lifelong consequences for the children who were raised in harsh family environments, as they become adults? Data indicate adverse medical, social and emotional outcomes. Considering the harsh environment as one of a continuing series of adverse childhood experiences (ACEs), especially early in life, consequences include premature death, inflammation, and cardio-metabolic, genetic and endocrine system dysfunction with excess healthcare utilization (58). Biomarkers for the earlier stressful circumstances, in addition to growth-related parameters of childhood that might be useful with adults include those related to inflammation (e.g., hsCRP); cardio/metabolic systems (e.g., BMI); genetics (e.g., telomere length) and the endocrine (e.g., HPA axis) systems (59).

## Stunting and Catch-Up Growth

Following the conclusion of WW II, a Medical Research Council (United Kingdom) project examined how the war and extreme food shortages affected the German civilian population, in particular children who were mainly short and thin for age in two orphanages in Germany (60). The focus of one study was a feeding experiment aimed at supplementing the barely adequate caloric intake with additional bread and jam, which was based on excellent growth noted at a different orphanage, also in Germany (61). The design followed a simple nutritional hypothesis: caloric deficit and its repletion. This was to be done by feeding two groups (houses) the same (albeit, meager) rations for the first six months and then adding extra bread, jam, and orange juice for the next six months in one of the houses. At the same time as the additional nutritional (6 months), one of the housemothers was transferred from the now “control” house to the “experimental” house, but in the process was permitted to take 8 of her “favorite” children with her. Of relevance, she was considered harsh and unsympathetic to all children except her favorites, such that the children (except those favored) were in constant fear over public reprimand by this housemother, specifically at meal times. The children in the “experimental” house, even with the additional nutritional supplementation, decelerated in growth compared to those in the “control” house who now had a different housemother (**Table 3**). It was concluded that some factor other than total energy (calories) counteracted the effects of the added nutrition. In addition, the housemother’s favorites from the original house who moved with her to the “experimental” house thrived slightly in excess of their original (“control” house) rate. It was thus noted that “…even the most perfectly planned nutritional investigation may be ruined by psychological factors, over which the investigator may have no control” [Widdowson, 1951, p. 1318] (60). Of interest, a Biblical reference [Proverbs (xv:17, KJV)] was noted in the context of this conclusion, “Better is a dinner of herbs where love is, than a stalled [*fatted*] ox and hatred”. In a recent review of this early post WWII “experiment”, it was concluded that “… psychosocial stresses due to harsh and unsympathetic handling could seriously curtail growth rates” [Arens and Ashwell, 2015, p. 12] (62). Thus, psychological stresses due to harsh and unsympathetic conditions can adversely affect linear growth.

**Table 3:** Weight change in German children refed in one of two houses.

Weight change in German children with refeeding following WW II			
Time	Weight (kg)		B
	V_1_	V_2_**	
(months)			
0–6	1.4	0.5	0.4
6–12	0.8	3.8***	2.0

*Data from Widdowson (61).

**Supplemental food at V_2_.

**Housemother from B to V at 6 mo.

***housemothers “favorites”.

In the second six months the housemother switched from B to V at the same time that the children in V received supplemental food. Data from Widdowson (61).

### Clinical Presentation

A survey of the medical records of >100 abnormally short children from a single endocrine clinic indicated that the stunted growth of approximately 50% of the children could be related to a systemic illness, including endocrine disturbances, but the short stature of 28 boys and 23 girls could not be attributed to a specific etiology (28). Among the latter children, all were <80% of average height for age and sex, and most had appropriate weight-for-height (weight age/height age X 100). Using this metric authentic, hypopituitary children were either of normal weight for height or overweight for height (>115% for this index). Skeletal age was also retarded in virtually all children and approximated their height age.

Nutritional histories of the children indicated feeding problems, often for extended periods including infancy, but generally not from birth. Most also had insufficient caloric intake for *chronologic* age, but protein intake was apparently in excess of the minimum required for normal growth. The latter, however, may not be valid in the context of chronic deficiency in total energy intake where protein may be used as an energy source.

A small subset of thin patients showed catch-up growth when placed on a diet adequate in energy and protein; by inference, the growth status of this group was attributed growth faltering due to total energy deficit. In contrast, approximately 40% of the children placed on a diet adequate in energy and protein did not experience a marked increase in weight and height. This was attributed to psychological and social stressors as many of the children had families with inadequate economic resources and in which “…the mother’s intellectual equipment was not sufficient to enable her to carry through adequate housekeeping and nutritional management of the child” [Talbot, 1947, pp 788–789] (28).

The investigators were pediatric endocrinologists and noted some similarities of their group of abnormally short children to children with hypopituitarism (28). The potential involvement of growth hormone deficiency was considered, although the discovery of hGH as a distinct protein was not established at the time of the study. To carry out a clinical “trial” methytestosterone (MeT) was substituted as the growth factor. Although all of the short children grew at an abnormally slow rate during the control period (growth faltering), all showed growth at normal or supernormal (CUG) rates while receiving MeT. Thus, the children were capable of growth when exposed to an “anabolic” agent, suggesting a relative deficiency of hGH during the control period and in turn implicating partial, but *transient*, functional hypopituitarism. In addition, the output of 17 ketosteroids increased during the period of MeT administration, implicating an increase in rate of production of 17-KS precursors by the adrenals and/or testes and an increase in endogenous pituitary activity (ACTH), since MeT is not metabolized and excreted as a 17 ketosteroid. The authors concluded, actually speculated, that the syndrome, i.e., short stature of the remainder of the 51 patients without a specific etiology may have represented functional hypopituitarism occasioned by limited caloric intake and significant psychosocial disturbances. Of note, correction of the emotional difficulties in a small minority of the children led to an increase in caloric intake followed by rapid weight gain and catch-up linear growth without additional therapy.

### Maternal Deprivation Syndrome (MDS)

Several syndromes are related to behaviors and psychosocial interactions associated with failure-to-thrive in infants and children. Maternal deprivation syndrome (MDS), or Type I emotional deprivation, has its onset in infancy, often very early, and involves a disordered infant/maternal interaction. A second syndrome, denoted as PSS, or type II emotional deprivation syndrome, generally has its onset beyond infancy. PSS involves bizarre behaviors, especially related to hyperphagia and food hoarding as well as absent GH secretion, and in some patients, subnormal responses to exogenous hGH. A third syndrome includes children who present with less severe failure-to-thrive and fewer of the distinct behaviors are prominent. This group shows proper GH response to stimulation testing and the children may respond to exogenous hGH. In the context of the preceding, a comment of Professor Robert M. Blizzard merits consideration:

“There are at least two different types of children with emotional deprivation-an older group who often have low plasma GH concentration and who frequently have no element of associated malnutrition. In general, these are children 3 years of age and above. There is another group who are less than 2 years of age, the type of patient Dr. Krieger in particular has studied. These are infants of mothers who don’t feed them, who have a very severe component of malnutrition. These children do not have GH deficiency” [Green, 1984, p. 40] (63).

The importance of chronological age as a key determinant of the physiological and behavioral phenotype was evaluated in two groups of children (**Table 4**): one, denoted as MDS included 9 patients 6/12 to 2 8/12 years and the second denoted as psychosocial (deprivational) dwarfism (*sic*) short stature (PSS), included 7 additional patients ages 3 5/12 to 10 2/12 years (15). The most relevant *pre-condition* for this distinction was the difference in fasting GH level, which was considered at the time of study for children within the two age distributions. All 16 met the criteria for a deprivational syndrome. Detailed clinical evaluation did not uncover an organic condition to which the growth faltering could be attributed. Confirmation of these deprivational syndromes depended on accelerated height velocity, subsequent neurodevelopment, and improvement in behaviors following removal from home. A sample of 8 chronically undernourished infants with growth faltering, but without a history compatible with MDS served as a control group for the MDS patients. All patients in all groups had normal or nearly normal endocrine system responses.

**Table 4:** Undernourished children evaluated for growth faltering based on their clinical presentation.

Maternal Deprivation Syndrome/Psychosocial Short Stature: Patient data^1^				
	No. of patients	Age range	Weight Percent for height	Length/height
		years		SD
MDS	9	0.5–2.7	76	-3.4
PSS	7	3.6–10.2	69.8	-5.4
Control^2^	7	1.0–3.0	similar^3^	similar^3^
(malnourished)				

^1^Data from Krieger and Mellinger (15).

^2^chronically undernourished, but without history of deprivation; similar to MDS in age, degree of linear growth failure and underweight relative to height.

^3^exact numbers not given but stated as “similar” to the MDS patients.

Given the observations for the three groups (**Table 4**), might the results be a manifestation of “toxic” stress, a chronic or frequent activation of the stress response from exposure to serious childhood adversity *without* adequate support or protection from adults (64)? It is possible that the infants and some older children experienced “toxic” stress. ACEs have serious long term outcomes in terms of delay in cognitive development and subsequent risk of cardiovascular and metabolic disease, but relatively little information is available on the type of stress, its intensity and/or duration, and the age at which these stressful events occur (65).

In addition to historical and clinical observations, the analysis of the growth curves of four children presenting with failure-to-thrive, severely delayed growth and delayed skeletal maturation could not be attributed to a specific organic pathology (66). The children also showed delayed motor and intellectual development associated with a disturbance of maternal behavior and family organization. The authors speculated on several possible, but non-exclusive mechanisms for the seemingly incongruent observations: inadequate nutrients, decreased appetite and feeding behavior, disordered family dynamics, a disorder of intestinal motility or absorption, and a disorder of the hypothalamus that might affect pituitary function.

## Endocrine System Alterations in Maternal Deprivation Syndrome and in Psychosocial Short Stature

### Malnutrition

The classic endocrine system response to the stress of malnutrition includes high basal GH and cortisol levels with a low circulating concentration of IGF-1. The elevated basal level of GH may increase even further following stimulation and reflects additional synthesis rather than impaired clearance (57). The biological activity of elevated GH levels likely:

- Facilitates lipolysis through activation of the hormone sensitive lipase in fat depots
- Enhances gluconeogenesis in the liver to avoid systemic hypoglycemia
- Decreases the catabolism of albumin

The very low level of circulating IGF-1 and growth faltering can be explained by resistance to the effects of hGH. Recent evidence suggests that the state of GH resistance in starvation may result from a decrease in signal transducer and activator of transcription (STAT) 5 phosphorylation mediated by fibroblast growth factor 21 (FGF-21) and/or Sirtuin1 (SIRT1) (67).

Serum IGF-1 concentration is very low in severe forms of protein-energy malnutrition (PEM), and its concentration is correlated with the BMI and the percentage of expected weight-for-age. Low protein and/or energy in the diet decreases IGF-1 production, while supplements of amino acids can increase IGF-1 synthesis (57). The selective decrease in IGF-1 synthesis as a manifestation of GH resistance can, in turn, decrease oxygen and energy utilization in general, and spare glucose and amino acids by reducing growth during energy deficit.

Basal cortisol levels are elevated in infants and children with malnutrition while the response of cortisol to ACTH stimulation is satisfactory. Testing with metyrapone showed a normal hypothalamic-pituitary-adrenal axis with PEM (57, 68). Malnutrition also increases nuclear glucocorticoid receptor abundance in marasmic children (69), which likely augments the action of circulating cortisol concentrations that mediate metabolic function:

- Increased lipolysis through potentiation of catecholamine action on hormone sensitive lipase to supply fatty acids as fuel
- Enhanced muscle protein catabolism to provide amino acids for hepatic gluconeogenesis and protein synthesis (e.g., albumin)
- Protection from hypoglycemia as an anti-insulin factor

### Infants With Maternal Deprivation

Variation in endocrine responses to emotional deprivation compared to protein-energy malnutrition in children was implicit in the premise of Krieger and Mellinger who believed that infants with maternal deprivation syndrome differed from those with PSS and malnutrition without maternal deprivation (**Table 4**). Blood sugar levels were normal in virtually all infants and most showed normal insulin sensitivity following insulin administration (**Table 5**).

**Table 5:** Endocrine data from undernourished children in **Table 4**.

Maternal Deprivation Syndrome/Psychosocial Short Stature: Endocrine Data^1^				
	Blood glucose	Growth hormone	Cortisol	17-ketogenic steroids
	Fasting/minimum	Fasting/maximal	Fasting/maximal	(fold baseline)
	(mg/dL)	(ng/ml)	(µg/dL)	
MDS	72/35	15/32	11/22	3.8
PSS	84/40	36/^2^	9/14.5	3.6
Control	86/41	13.4/45.6		4.8
(malnourished)				
Control		5.4 ± 3.9^3^	14 ± 7.4/23 ± 7.0^4^	
(normal)				

^1^Data from Krieger and Mellinger (15).

^2^abnormal in 5.

^3^fasting only.

^4^data from Bertrand, et al. Ann Endocrinol 1963; 24:881-887.

### Growth Hormone/IGF-1 and Hypothalamic-Pituitary Adrenal Axes

GH concentrations in the fasted state at the start of the insulin tolerance test were measurable, but variable (**Table 5**) and tended toward the elevated levels. Infants with PSS had lower basal levels of GH, and GH release to insulin-stimulated hypoglycemia was inadequate in 5 of 7 with PSS (**Table 5**). In three infants, 2 with MDS and 1 with PSS, the GH response was inadequate, but sufficient hypoglycemia was not attained. Since all infants had fasting levels >7 ng/ml, they were not considered GH-deficient. In contrast, all other infants had an adequate GH response to the stimulus.

Based on the preceding data, the 5 oldest patients, all with PSS, were considered GH deficient. Baseline samples were obtained in the first few days of hospitalization, and 3 of the 5 infants with apparent GH deficiency had GH levels >8.8 ng/ml and were thus not considered GH deficient by this criterion. The three infants with *apparent* GH deficiency were placed in a more nurturing environment and subsequently experienced CUG and had normal GH release during this interval. The other two patients returned home, but had relapses and were readmitted to the hospital; insulin tolerance tests indicated GH deficiency. Although these data have limitations in that some infants labeled PSS did not have documented GH deficiency, their responses to change in domicile suggest that from the pathophysiologic context, they were GH deficient, or perhaps GH and/or IGF-1 resistant.

### Children With Psychosocial Short Stature

Thirteen children (3 females and 10 males, ages 3.3 to 11.5 years) who had growth faltering simulating idiopathic hypopituitarism and who were evaluated over 6 years presented a clinical picture with many features that were not typical of the large number of hypopituitary patients evaluated at the Johns Hopkins clinic (25). The historical clinical features included feeding difficulties in infancy, persistent sleep problems, parents who often showed significant psychological disturbances, and a *marked* improvement in growth status with foster placement. All children presented bizarre behaviors, but had relatively normal physical examinations, although a majority had notable abdominal protuberance. The behaviors included polyphagia, polydipsia, gorging and vomiting, hoarding food, eating from garbage pails and drinking from toilets. The children also did not sleep well, roamed at night, and showed many abnormal behaviors including apathy, anxiety, irritability, temper tantrums, shyness, and self-injury. Their overall development including speech and cognition lagged relative to their age peers. Finally, interactions with their primary caregivers suggested that the latter may have had depression, anxiety or personality disorder, or experienced domestic violence.

The 13 children were admitted to Johns Hopkins Hospital for detailed metabolic and endocrine studies, then sent to a nearby convalescent hospital for 3.5 to 12 months and subsequently re-admitted to the hospital for further evaluation. Eleven of the 13 children were originally evaluated for short stature and 8 had steatorrhea, while the other 2 were referred for behavior problems (**Table 6**).

**Table 6:** Evidence for hypopituitarism *before* catch up growth in 13 children with emotional deprivation.

Evidence for Hypopituitarism *before* Catch-up Growth^1^	
**Sign or Symptom**	**Number**
Short stature	13/13
Delayed bone age	10/13
Abnormal thyroid function	4/13
Abnormal HPA axis	12/13
Growth hormone deficient	6/8^2^

^1^Data from Powell, et al. (25).

^2^only 8/13 tested.

All of the males had delayed bone age, while bone ages of girls were near normal or normal. Most of the general laboratory and imaging studies were within normal limits, although several children were anemic. Two major clinical observations were noted: first, many of the bizarre behaviors related to drinking and eating either disappeared immediately or persisted for only a few days, and second, all children became less withdrawn once hospitalized.

Results of the detailed endocrine testing prior to admission to the convalescent hospital and after prolonged stay at the convalescent hospital are summarized in **Tables 7**, **8** (26). No medications were administered and no special psychological therapy was given to spur the reversal of pituitary function. During initial hospitalization, all gained weight, which continued at the convalescent hospital. The rapid increase in linear growth was striking and indicated significant CUG in all. The average increase was about 1.65 cm/month, more than three times the average rate of normal growth, approximately 0.5 cm/month. The magnitude of the height velocity was not compatible with permanent hypopituitarism (GH deficiency). Nevertheless, and of importance, the majority of children had a marked and very rapid decrease in height velocity when discharged to home from the convalescent hospital in contrast to the very rapid and marked increase in height velocity upon entry to that facility. The rapidity of the responses was not compatible with a nutritional hypothesis for growth faltering.

**Table 7:** Hypothalamic-pituitary adrenal responses to metyrapone and ACTH in children with psychosocial short stature before and after convalescent hospital stay.

Hypothalamic-Pituitary-Adrenal Axis^1^	
Pre-growth period	
9/13	Low at baseline
12/13	Inadequate response to metyrapone
6/7	Normal to ACTH stimulation
After growth period	
10/10	Normal at baseline
8/10	Adequate response to metyrapone^2^

^1^Data from Powell, et al. (26).

^2^one became normal 3 ½ years after growth resumed.

**Table 8:** Growth hormone response to hypoglycemia in children with psychosocial short stature before and after convalescent hospital stay.

Growth Hormone Response to hypoglycemia, children with PSS^1^	
Before growth	After growth
6/8 no response	6/6 adequate response
(all samples <5 ng/ml)	(peak 17–52 ng/ml)
2/8 adequate response	
(16 and 36 ng/ml)^2,3^	

^1^Data from Powell, et al. (26).

^2^one in hospital for 3 weeks before test done.

^3^one growing at a normal rate for the two prior years.

In summary, the striking features of the 13 children included an adverse family/home environment, bizarre personal and social histories, and perhaps a protuberant abdomen. This history separates the group from many other children presenting with short stature and growth faltering. The overall clinical picture suggested that emotional factors, malabsorption, inadequate nutrition, and hypopituitarism (with a likely disturbance of the hypothalamic-pituitary GH/GF-1 axis, including GH resistance) contributed to the growth faltering. However, some of the children may have multiple factors which lead to a final common pathway underlying growth faltering.

None of the 13 had clinical hypothyroidism before admission to the convalescent hospital, although many had markedly delayed bone ages without epiphyseal dysgenesis which is common with long standing hypothyroidism (26). Thyroid hormone testing and TSH determinations, using more indirect methods than available at present, did not indicate significant thyroid disease. Adrenal function was evaluated by baseline 17-hydroxycorticosteroids in the urine and stimulation of pituitary ACTH with metyrapone; 9 of 13 baseline adrenal function studies and 12 of 13 metyrapone studies were abnormal (**Table 7**) and indicated dysfunction of the hypothalamic-pituitary-adrenal axis. The excretion of a water load, a test of adrenal (glucocorticoid and mineralocorticoid) function, indicated 2 of 11 children with abnormal tests compatible with adrenal insufficiency. ACTH stimulation tests were normal in 6 of 7 children tested (**Table 7**). The fasting blood sugar and oral glucose tolerance tests were normal in all data (not shown). Eight of the 13 children were tested for GH deficiency with an insulin tolerance test. Six of the eight had no or low stimulated GH levels when the blood glucose level decreased by at least 50%, but in two children the stimulated level was normal. Of the latter, one had been at hospital for three weeks before the test was performed and the other had a normal height velocity for the preceding 2 years (**Table 8**).

After the stay at the convalescent hospital, 11 of 13 children were re-admitted for repeat evaluation. All had normal thyroid function and 17-hydroxycorticosteroid baseline tests, but the metyrapone stimulation test remained abnormal in 8 of 10 children (**Table 7**). For GH, 6 of 6 children who secreted no GH in the original test, now had a normal stimulated GH level (**Table 8**), and none showed abnormal sensitivity to the hypoglycemic stimulus.

It appeared that hypopituitarism, at least the GH deficiency, was reversible and likely due to psychosocial factors given the rapidity of change in height velocity, decreased at baseline or when discharged to home, but accelerated upon entry to the convalescent hospital. One patient tested after 3 weeks in an acute care hospital likely showed the rapidity of the recovery of GH secretion in a more nurturing environment. Although basal adrenal secretion normalized during the interval at the convalescent hospital, response to the stimulus, metyrapone, did not recover as rapidly; however, none of the children had signs or symptoms of adrenal insufficiency. Others have noted that it may be as long as 2 years for complete recovery of the hypothalamic-pituitary-adrenal axis (70).

In a series of short children, ages 3 years 6 months to 10 years, evaluated for GH deficiency by insulin tolerance testing, 9 met the criteria for PSS (71). Seven of the 9 had normal values (**Table 9**), although the precise timing of testing relative to hospitalization was not indicated. Nevertheless, it was concluded that the 10 control subjects and 7 of the 9 with PSS had normal responses to the insulin tolerance test. These data highlight the variability in the pituitary response to GH provocation among children with seemingly the same psychosocial and growth criteria, and also emphasize the spectrum of hypothalamic and pituitary responses to the same stressors. Nevertheless, variability in testing, especially the day of the test relative to removal from the home may be important.

**Table 9:** Hypoglycemia-induced growth hormone stimulation in 9 patients with PSS and control children.

Growth Hormone Stimulation Tests^1^		
	Control	PSS
Age (years)	3 7/12–13 1/12	3 6/12–10 0/12
GH (ng/ml)		
Fasting		
mean	8.3	2.4
range	2.0–14.7	1.0–4.3
stimulated		
mean	12.4	10.9
range	3.4–28.4	1.0–19

^1^Data from Kaplan, et al. (71).

The adrenal axis was evaluated by measuring plasma cortisol at baseline and following insulin-induced hypoglycemia. Most were within normal limits (**Table 9**), but three with PSS had a small increment and one had no increment in cortisol concentration. All were in the group with apparent GH deficiency. Virtually all of the metyrapone tests also showed the expected increment in 17-ketogenic steroids (**Table 9**).

The preceding data and the observations of Powell and co-workers are not easy to reconcile. In general, the age groups likely represented different metabolic mechanisms with infants more likely to present largely as energy deficient. As shown by Krieger and Mellinger (15), when given sufficient energy and proper attention and care, infants can grow without additional therapy, either pharmacologic or psychological On the other hand, the PSS group is more heterogeneous, spanning a spectrum of similarity to infants at one end to hypopituitary (GH deficient) children at the other. The data for the PSS group in **Table 4** (72) indicate less severity compared to the patients in **Tables 6****–****8** (25, 26). It is not clear, however, why so many of the former had as high basal and stimulated GH levels as protein/calorie malnourished infants and children (7, 56, 57). Basal GH levels were elevated in children with nutritional marasmus and kwashiorkor; the levels declined when protein was added to the diet, but remained high on energy repletion without protein (56). Similarly, high GH and cortisol levels, but low insulin and IGF-1 levels were noted in malnourished Egyptian children; the levels reverted to normal (diminished GH and cortisol, and augmented IGF-1) during a three week re-feeding program (7, 57).

Underfeeding due to neglect is not uncommon among infants and children with deprivation syndromes (emotional and food). Food deprivation likely causes growth faltering and endocrine abnormalities. Pituitary *hyper*function (elevated GH and cortisol levels) with malnutrition may progress to include hypothalamic insensitivity, and in severe cases to pituitary *hypo*function, especially of the GH and adrenal axes.

### Other Investigations

The clinical evaluations of 16 children, 2 to 16 years, presenting with growth stunting without an apparent physical cause have been reported (73). Most were of average length and weight in early infancy, but feeding difficulties were common, including several who had eaten well during previous hospitalizations for growth faltering or failure-to-thrive. Family histories, feeding habits, and family relationships were consistent with PSS. Serum GH responses to insulin-induced hypoglycemia were marginally low in only one child, although the timing of the sample during a two week hospitalization was not specified (**Table 10**).

**Table 10:** Growth hormone dynamics in children with emotional deprivation syndrome at evaluation.

Growth Hormone Dynamics in Children with Psychosocial Short stature				
Subjects	Age range	GH testing		Reference
(n)	(y-y)	normal	Abnormal	
16	2–16	15/16	1/16^1^	Apley et al. (73)
3	4–13	3/3	0/3^2^	Howse et al. (74)
1	6	^3^	^3^	Stanhope et al. (75)
1	3.3–11.5	^4^	^4^	Powell et al. (76)
1	5 8/12–6 1/12	0	2/2^5^	Mouridsen et al. (77)
9	3 1/12–10 0/12	7/9	2/9	Kaplan et al. (71)
3	6–12.7	0/3	3/3	Holmes et al. (78)
16^6^	3.8–13.7	1/16	15/16	Skuse et al. (51)
6		5/6	1/6	D’Ercole et al. (79)
1^7^	18–27	5/6	1/6	Magner et al. (47)
11	2.2–13.5	^8^ 11/11	^8^ majority	Albanese et al. (80)
1	5.7; 9.3	^9^	^9^	Powell et al. (26)
7	3.6–10.2	1/7	6/7	Krieger et al. (15)

^1^marginally low.

^2^5 h integrated level during sleep.

^3^19 integrated level increased from days 1 to 6 to 18 d of hospitalization.

^4^sleep related GH levels 4.4 and 5.6 ng/ml on days 5&6 of hospitalization; 30 ng/ml day 40.

^5^Patient tested on two separate hospitalizations during PSS-compatible history.

^6^All hyperphagic.

^7^Multiple tests over 9 years; first test abnormal at age 18 years.

^8^1^st^ day spectrum of abnormal patterns; increased amounts released by day 10 and further increase day 40.

^9^Two separate evaluations. Both diminished GH release day 1 or 2; normalized by 5 or 6 weeks in hospital.

Low peak GH levels were noted in three children with PSS along low 5 h integrated levels of GH, which were in the range of children with documented hypopituitarism (74). The GH response to hypoglycemia was also subnormal (**Table 10**).

A case study of a 6 year old boy with reversible GH deficiency secondary to PSS indicated an accelerated rate of growth on removal to a more favorable home environment, thus confirming the presumptive diagnosis (75). GH status was evaluated three times during initial hospitalization with GH profiles obtained over 19 h. Levels of GH increased from days 1 to 6 to 18 in an amplitude-modulated manner, and a more physiologic sleep-related initial peak was noted (**Table 10**)

Eleven children with growth failure and psychosocial deprivation were clinically evaluated (80). Three sets of 18 h GH profiles were done on all but one child during a 3-week initial hospitalization. The GH profile on the day of admission was lower and variable in terms of frequency and amplitude of GH release. This pattern was reversed toward normal in the ensuing two GH profiles with an increase in GH secretion in a pulse amplitude modulated manner (80). The results highlighted a reversal of the deficiency on removal from the inciting environment, even in the hospital, within 2 to 3 weeks (**Table 10**).

A study of the relationship between sleep and growth in patients with PSS in 27 patients 2 to 16 years, noted a relationship between sleep quality and rate of growth (81). When sleep was considered “good”, patients grew at a rate of 1.04 ± 0.38 cm/month, but if considered “poor”, the growth rate declined to 0.34 ± 0.13 cm/month. Although GH concentrations were not measured, the peaks and valleys of height velocity appeared to be due to hGH deficiency, especially related to the onset of deep sleep (Stages III and IV). In a child with PSS admitted to the hospital on two occasions, a lower sleep-related peak GH release was noted during normal slow wave sleep on the 5^th^ and 6^th^ nights of hospitalization (4.4 and 5.6 ng/ml), compared to 30.0 ng/ml on day 40, which was also correlated with the onset of the initial slow wave sleep cycle (76). Overall, the results suggested a good correlation among sleep, time in the hospital, GH release to a hypoglycemic stimulus, and robust CUG (**Table 10**). The importance of sleep architecture was also noted in a patient diagnosed with PSS who had very little slow wave sleep at the beginning of hospitalization (82). Although growth hormone levels were not measured during the study, a normal level was noted on day 9.

The preceding studies suggest a diminution of slow wave sleep in patients with PSS. These decrements may be causal for low GH release, which most commonly occurs with the onset of the first episode of SWS. As children acclimate to a new and more nurturing environment, whether it be during initial hospitalization or after changing domiciles, there is an increase in SWS, GH secretion and over the longer term normal or CUG in height.

In a single patient with a classic history of PSS, the growth curve showed minimal linear growth over an approximately 3 year interval, but was followed by robust CUG at age 7 years after placement in a more nurturing environment (77). While in a stressful foster home, treatment with hGH did not result in CUG or any significant change in the very low baseline growth rate. While at the foster home, the patient had 2 clonidine tests which were consistent with GH deficiency. When moved to a more nurturing environment, GH release and IGF-1 levels returned to normal, CUG occurred and he showed substantial social maturity, intellectual growth, academic achievement and improvement in language skills. The anti-social and bizarre behaviors.

A subset of the type II children with growth-faltering, behavioral disturbance and psychological stress in addition to hyperphagia presented with normal weight (51, 55). After removal from the stressful home to a more nurturing environment, the hyperphagia and GH deficiency resolved spontaneously in the majority. Based on symptom profile, physiology, associated intellectual impairment, and familial aggregation, it was proposed that the hyperphagic phenotype had predictive and discriminant validity for growth hormone deficiency.

Skuse and colleagues evaluated 51 consecutive patients (29 boys and 22 girls) who were hospital-referred for growth failure unrelated to organic pathology, but who came from “stressful” homes. An additional 32 short children from the community (The Wessex Growth Study, 32 of 140 eligible short children; one moved to the hyperphagic group) were evaluated as a control group. Of the referred group 28 were hyperphagic (with an additional one from the community group) and 23 were non-hyperphagic. Subsets of hyperphagic but with a normal BMI, characteristic symptoms related to food (gorging and vomiting, stealing food, nocturnal searching for food) and polydipsia, and non-hyperphagic children were subsequently evaluated over a three week hospitalization to test for spontaneous changes in GH secretion or to stimulation testing (none to both groups). Two 18 h GH profiles were obtained, the first starting within an hour of admission and the second at a mean of 18 days later. Sixteen of 24 hyperphagic children had initial standard provocation tests on the day of admission to hospital. Fourteen (88%) were initially considered GH deficient (peak GH < 20 mU/L; *n.b.* 1 ng/ml ≡ 3 mU/L) (**Tables 10**, **11**). Ten of the 16 were retested and all showed an increase in GH response to the stimulus. The mean peak increased from 8.9 ± 1.52 to 37.2 ± 8.4 mU/L, compatible with the neurosecretory profiles on the other 5 hyperphagic children (**Table 11**). Of the non-hyperphagic children evaluated (approximately 75% were anorexic with low BMI), the mean peak increased only from 22.6 ± 8.9 to 26.7 ± 6.3 mU/L indicating GH sufficiency in both tests (**Table 11**). Clear *reversibility* was shown in only one patient. Of specific interest, it was noted that, in contrast to the rapid resolution of the appetite disorder in the hyperphagic children, the poor appetite noted at intake persisted in the anorexic children throughout the hospitalization (51). The marked difference in the behavioral criteria noted above strongly identified the two subsets. The authors note the predictive and discriminant validity of the symptom profile. The mean IQ scores of the groups: hyperphagic 76.3 ± 20; non-hyperphagic 93 ± 31 and community (comparison) 104 ± 15.

**Table 11:** GH Testing in Hyperphagic and Non-hyperphagic Children with Emotional Deprivation.

GH Testing in Hyperphagic and Non-hyperphagic Children with Emotional Deprivation^1^				
	GH profile^2,3^		GH provocation^4^	
	Mean, mU/L		Mean peak, mU/L	
	Hyperphagic	Non-hyperphagic	hyperphagic	Non-hyperphagic
Early^5^	3.0 ± 0.6	2.43 ± 0.54	8.9 ± 1.52	22.6 ± 8.9
Late^6^	6.3 ± 0.54	2.82 ± 0.8	37.2 ± 8.4	26.7 ± 6.3

^1^Data from Skuse, et al. (51).

^2^n = 5 hyperphagic; n = 5 non-hyperphagic.

^3^1 ng/ml ~ 3 mU/L; cut-off for GH deficiency considered at 20 mU/L.

^4^n = 16 hyperphagic; n = 16, non-hyperphagic.

^5^first day of hospitalization.

^6^18^th^ day of hospitalization (average).

One could differentiate the pathophysiology of the two clinical groups by noting that the children in the non-hyperphagic group were stunted as a consequence of chronic nutritional deficiencies. The hyperphagic group were considered stunted as a consequence of hypopituitarism at the time of first evaluation. The data would argue that these are two separate conditions based on symptom complex and pathophysiologic mechanisms, rather than a single condition along a severity spectrum as noted above.

Based on the results, the primacy of stress was questioned since one of the control groups included a similar number of *equally stressed* children, but who had grown normally (55). Perhaps the discrepancy between the groups reflects the broad spectrum of sensitivity of the hypothalamic-pituitary axis rather than distinct pathophysiologic entities (53). The qualitative aspects of the individual stressors and/or the responsiveness of individual children, whether genetic, epigenetic or environmental, may be an additional source of variation. Some of the same authors also presented follow up growth data. All hyperphagic and non-hyperphagic children had initial CUG following intervention (removal from the domicile or intensive social services) with height velocity SDSs increasing after the intervention from -1.4 ± 1.4 to +1.8 ± 1.8 SDS in the former and from -1.3 ± 1.1 to +1.0 ± 1.2 SDS. All in the non-hyperphagic group, whether receiving GH or not, received social services interventions and increased HV from -1.7 ± 1.3 to +1.2 ± 1.6 SDS. A subset of the original sample had short young adult heights (about -2.4 SD) compared to a mid-parental target height of -1.9 SD (p < 0.001); the adult heights were within the mid-parental target height *range*, but at the lower end (54).

Among 6 patients with PSS and associated behavioral abnormalities, 5 had subnormal GH responses to stimulation tests and low circulating levels of IGF-1 (79). With hospitalization, marked positive changes were noted in personality characteristics before increases in IGF-1 levels and a striking acceleration of linear growth following change of domicile, highlighting the correlation with increased GH secretion (**Table 10**). IGF-1 levels (measured as somatomedin C activity) of 3 children with PSS were 0.20 ± 0.02 U/ml, similar to children with documented GH deficiency (78). With exogenous hGH, IGF-levels increased to 0.81 ± 0.2 U/ml, and two of the children had CUG during a short-term removal from the deleterious environment. With continued therapy, but living under the same conditions as they did initially, the children did not show accelerated growth leading to the consideration of IGF-1 resistance as a potential mechansim (**Table 10**).

### Skeleton

Radiographic indicators compatible with very slow growth include multiple lines of arrested growth in the long bones (83, 84) and widening/splitting of the cranial sutures associated with very rapid growth in younger patients at the convalescent hospital (85).

### Onset at Adolescence

Although PSS usually occurs during childhood, severe psychological trauma during adolescence can occasionally trigger a similar reaction as noted in several boys with signs and symptoms of PSS associated with the onset of pubertal maturation later than 16 years (46). The index patient was a 16 year old boy initially evaluated 6 weeks after removal from the inciting environment. His history indicated weight gain (about 5 kg), but he had a height age of 8 years and 6 months and a bone age of 11 years and one month, and showed no signs of pubertal maturation. After 10 months in a more nurturing environment, he grew 10 cm and gained more than 9 kg. Early indicators of pubertal maturation were apparent. At 18 years, height age was 13 1/2 years and bone age was 15 ½ years. He was fully sexually mature (46). Under somewhat similar circumstances, two adolescent females in emotionally stressful situations had height velocities that were markedly decelerated before evaluation (86). When the environment became less stressful, a normal pattern of growth and maturation was soon noted in both.

A detailed report of a male 17 years and 8 months of age presenting with diminished growth and lack of progress in pubertal maturation indicated a history of no problems until about 12 years at which time his height and weight approximated the 50^th^ and 10^th^ centiles, respectively (47). He had a disagreement with his step-father and verbalized that he wished that the step-father would die. The next day, the step-father fell from a roof, was severely injured and required prolonged hospitalization during which the patient (step-son) was not permitted to visit. Subsequently, an inadvertent death notice was received at home by the patient who viewed himself as responsible. The youngster essentially stopped eating and lost weight to well below the 1^st^ centile. At 12 years and 6 months, he had a fasting blood sugar level of 24 mg/dL and required tube feedings because of refusal to eat. He was hospitalized a psychiatric facility and then at a facility for emotionally disturbed adolescents. During this time, he experienced no weight gain or linear growth and showed no signs of pubertal maturation. At the time of referral and evaluation at 17 years and 8 months, his height was well below the 1^st^ centile and the history was as noted, although he stated that his appetite was a little “better”. There was no history of physical abuse and the review of symptoms suggested no reference to the endocrine system other than failure of growth and pubertal maturation. He was thin and pale and appeared very much younger than his chronological age. The genitals were pre-pubertal, bone age was between 13 and 13 ½ years, and blood chemistry determinations were normal. The response to metyrapone was normal, and the GH response to an arginine-insulin tolerance test peaked at 6 ng/ml at a time when the testosterone level was 100 ng/dL (**Table 12**). A year later and with no specific therapy, he had grown 1.5 cm, but peak GH was now 15 ng/ml and morning testosterone levels were between 50 and 100 ng/dL. GH therapy was begun in the next year and height velocity increased to 4 cm over 8 months. GH therapy was stopped after one year without much change in height velocity, although the testosterone level increased to 170 ng/dL. Within another year, the testosterone level increased to 550 ng/dL and maturity status was late pubertal. When re-evaluated at 27 years and 8 months (more than 6 years after the previous exam), he had grown an additional 10 cm to an adult height of 171 cm (**Table 13**).

**Table 12:** IGF-1/Somatomedin C responses of children/adolescent with emotional deprivation.

Somatomedin C/IGF-1 levels^1^			
**Number**	**pre**	**post**	**reference**
6	5/6 decreased	6/6 normal	D’Ercole et al. (79)
3	0.20 ± 0.02 U/ml	0.8 ± 0.2 U/ml	Holmes et al. (78)
1	6/8 low; 2/6 marginal		Mouridsen et al. (77)

**Table 13:** Longitudinal patient and endocrine data for an early adolescent who underwent severe psychological trauma at age 12 years.

Patient data^1^						
CA^2^	HA^3^	BA^4^	HV^5^	GH_peak_	T^6^	Therapy
(years)	(years)	(years)	cm/year	ng/ml	ng/dL	
17 8/12	11 4/12	13–13 1/2	~1	6	100	none
18 6/12	11 9/12	–	1.5	15	50–100	none
19 4/12	12 0/12	15	2.4	13	82	begin growth hormone
20 0/12	12 8/12	15	6	10	170	Continue growth hormone
20 7/12	13 0/12	14–15	9	–	~500	Discontinue growth hormone
21 1/12	13 4/12	14–15	5	22	–	none
27 6/12	15 0/12	–	10.5^7^	27	–	none

^1^Data from Magner et al. (47).

^2^chronological age.

^3^height age.

^4^bone age.

^5^height velocity.

^6^testosterone.

^7^10.5 cm over ~ 6 years, but do not know when linear growth ceased.

This male adolescent initially tested as GH deficient and continued to grow very slowly even though later tested as GH sufficient. He was eventually administered an adequate dose of rhGH, but had a growth response as if GH insensitive. Spontaneous pubertal maturation occurred at 20+ year’s age and he achieved a normal adult height, weight, and sexual maturity. The reversible deficit in GH in this patient and in those with psychosocial short stature was not likely due to nutritional factors, *per se*. This young man represented a rare instance in which severe psychological trauma likely altered regulation of certain anterior pituitary hormonal axes, either at the level of the hypothalamus and/or at higher cerebral centers (47).

### Growth Hormone Resistance

Some evidence also suggests resistance to administration of hGH. This was noted in 4 of 100 patients with GH deficiency who met the criteria for PSS, although a milder form compared to earlier reports (25, 87). The patients did not have accelerated growth during treatment with hGH and all remained at home during the therapeutic trial. The children did show a release of GH during stimulation tests, but the tests were done days after initial hospitalization.

A single 6 ½ year old girl with a history of emotional deprivation was diagnosed with GH deficiency secondary to emotional deprivation (88). The patient had a subnormal height velocity compared to that of a hypopituitary child treated with hGH. When she left home for a more nurturing environment, she grew at a rate of ~16 cm/year without hGH treatment.

The adolescent described above (47) also had several trials with hGH but showed little change from baseline height velocity. It was only several years after hGH therapy was discontinued and when he was near adult pubertal status that a remarkable change in psychosocial status apparently contributed to spontaneous onset of adolescent growth spurt. Several patients noted by Holmes and co-workers had normal IGF-1 concentrations during treatment with hGH, but diminished growth, a possible indicator of IGF-1 resistance (78). Inflammation and malnutrition can also result in GH resistance as suggested in high sensitivity C-reactive protein (hsCRP) and GH, IGF-1 and IGFBP-3 levels in malnourished and often infected infants and children in Fortaleza, Brazil (11). The hsCRP levels were positively related to GH levels, but negatively related to IGF-1 and IGFBP-3 levels, suggesting a state of GH resistance. After adjusting for inflammation, IGF-1 and IGFBP-3 levels were positively associated and GH levels were negatively associated with Z-scores for height and weight. The data indicate the complex interactions among inflammatory processes associated with infection and malnutrition, malnutrition *per se*, several endocrine axes, the growth plate *via* alterations in chondrogenesis, and perhaps local growth factor signaling (11, 89).

## Dénouemont

That energy deficit and several psychosocial stressors can affect the growth, maturation, and development of children is axiomatic with multiple examples in the primary data noted above. The timing of the events (energy deficit, stressors) span fetal life to full maturity, the degree of deficit or stress, and the length of its primary effect can influence the life history of the individual.

Examples abound from the delimited energy intake of pregnant women during the Dutch Hunger Winter spanning November 1944 to May 1945 due to the German blockade of food supplies to the western portion of The Netherlands (90) to the untargeted epigenetic analysis comparing same sex siblings of those affected *in utero* which showed 6/181 differentially methylated regions associated with exposure to famine as the insulin receptor and carnitine palmitoyl transferase 1A were significantly associated with birth weight and LDL cholesterol levels (91). Depending whether the energy deficit and concomitant stresses started in the first, second, or third trimester and carried through to delivery, outcomes varied. These children have now been followed for more than 5 decades. Those exposed in the first trimester had a considerably lower probability of employment, perhaps due in part to diminished cognitive abilities and an increase in cardiovascular diseases, not unlike small-for-gestation infants and the Barker hypothesis (92). Those exposed in the second or third trimesters had an increased number of hospitalizations, a measure of diminished general health (90). The malnourished, stunted children may be of the same body size as those with emotional deprivation, but the former have different psychosocial abnormalities compared to those with MDS or PSS. In the latter, the psychosocial/maternal deprivation occurs first with deterioration of cerebral function leading to the psychosocial disturbance. In malnourished children, inadequate nutrition (severe, prolonged, or both) occurs first and has a negative influence on subsequent developmental progress and psychosocial manifestations. Although all malnourished infants/children have low energy intake, those with low quantity and quality of protein intake (kwashiorkor) have more psychosocial abnormalities compared to those receiving low protein quantity with reasonably good quality of protein (marasmus). It has recently been recognized that re-feeding with greater amounts of protein may be a prominent factor for obesity in these children several years later (93). However, for those with stunting due primarily to malnutrition, maternal and broader social neglect due to poverty and associated life stresses (ACEs) may synergize with the poor nutrition in the development of stunting and also a less robust response to nutritional rehabilitation at home.

The hypothesis for the role of fetal factors and growth on subsequent life course outcomes has been further expanded to the concept of developmental origins of health and disease (DOHaD) accounting for the impact of postnatal growth and other factors, perhaps psychosocial stressors, during the first years of life (92).

Observations from the 1944 to 1945 famine in The Netherlands were complemented by those accompanying the siege of Leningrad between 1941 and 1944. The latter population was previously malnourished and remained malnourished after the siege. The infants did not have accelerated weight gain. Glucose metabolism in the Dutch revealed insulin resistance, but was not noted in Leningrad population, suggesting that an overabundance of nutrition post-natally may have a lifelong influence on diminished health (92). Not only would the fetus be “small”, there were effects that were transmitted through the life history of the next generation, likely through epigenetic mechanisms as “codified” in the original Barker hypothesis (92). In other words, F_1_ embryos growing in the F_0_ pregnant mothers already contained germ cell precursors that would generate F_2_ individuals. Thus F_0_, F_1_, and F_2_ cells are exposed to the initial stressors.

The issue of nutrition (total energy deficit *versus* psychosocial stress, or the combination of the two) is not completely clear, but the observations of Whitten and colleagues (9) and Krieger (72) favor a nutritional hypothesis for the younger infants. The separation by age group has been confirmed in part from a study of the health of children adopted within and from Romania (94). Observations of 34 children ≤14 months of age and 31 children >15 months of age who had been adopted (mainly from orphanages) within Romania suggested gross deprivation and a lack in sensory or auditory stimuli, physical stimulation and opportunities for forming attachments to caregivers. All were short-for-age, but the weight-for-length was within normal limits, eliminating a purely nutritional hypothesis. Growth faltering was intensified with length of stay in the institution with the children losing about 1 month of linear growth for each 3 months in the orphanage; however they were able to maintain body weight proportional to the height increase. The infants had general failure-to-thrive and growth faltering, and the older group had progressive short stature, but with more severe developmental delays. These observations are not unlike the two (age) groups of emotionally deprived children described above. Several studies have evaluated growth failure in international adoptees to stable families in the United States and noted similarities to emotionally deprived children (**Table 14**). Although not as growth retarded as the Romanian children (and certainly not randomly chosen for adoption from the large institutionalized group), most did show catch-up growth within the first six months of the new environment (95, 96). The more important factors associated with increased height, weight, and growth in head circumference were younger age at adoption, shorter height, and increased caloric intake. As noted for children who had moderate-to-severe emotional deprivation, some international adoptees, especially from institutions, had similar growth and intellectual gains although the etiology is likely multi-factorial. The emotional and/or nutritional deprivation precludes adequate environmental stimulation and nutrition for normal physiologic growth and the stress of psychological harassment underlies abnormal circadian rhythms and suppression of GH release (97). A meta-analysis of international adoptees noted large lags in height, weight, and head circumference at placement with virtual complete catch-up in height and weight, but incomplete catch-up in head circumference (98). Corresponding data do not exist for a similar group with emotional deprivation, but the remarkable CUG and developmental advancements very early after removal from the inciting environment make the outcomes for these children similar to those noted in adoptees, if the former can subsist in a stable environment.

**Table 14:** Demographic and auxologic data on adoptees and emotionally deprived children.

Demographics and growth parameters^1^			
Variable	IA^2^	US deprivation^3^	Control^4^
	n = 15	n = 17	n = 28
Age (months)	73 ± 3	64 ± 7	67 ± 4
Height Z	-0.5 ± 0.21	0.03 ± 0.32	0.29 ± 0.23
Weight Z	-0.59 ± 0.22	0.10 ± 0.28	0.01 ± 0.26
Wt for Ht Z	-0.31 ± 0.28	0.19 ± 0.22	-0.24 ± 0.21
BMI Z	0.32 ± 0.23	0.21 ± 0.23	-0.11 ± 0.21

^1^data from Miller, et al. (97).

^2^international adoptees.

^3^US children evaluated for emotional neglect.

^4^children from 2 parent homes, without history of abuse, neglect, or adoption.

Children who experienced institutional care during infancy have impaired responsiveness of the HPA axis to psychosocial stressors (99, 100). The blunted cortisol response can carry over into adulthood even if the children are placed in supportive, well-resourced homes. However, the peri-pubertal period may be a second epoch of plasticity to permit the HPA axis to recalibrate to a physiologic state (101). Given the similarities between institutionalization and emotional deprivation prior to the peri-pubertal period, one might consider this an important “window” for such children to regain disordered HPA axis function to psychological stressors (101). One might summarize that adverse events that lead to embryonal, fetal, or neonatal epigenetic changes (marks) are responsible for altering the mechanisms of growth, metabolism, cognition, and behavior of the infant and these alterations persist for the remainder of the individual’s life.

For children and the occasional adolescent, a singular nutritional hypothesis does not seem tenable. The rapidity of change in height velocity, whether increasing as the toxic environment is left for a more nurturing one, or decreasing as one returns to the original environment does not support a unique nutritional hypothesis, but suggests the primacy of a psychosocial one. The observational studies after WW I and II show that, in the main, children from stable backgrounds before the toxic stress (in these cases war and starvation) showed catch-up growth to pre-stress levels, although a large number of German orphans and those from unstable pre-war backgrounds did not show this type of growth and gained weight in excess of height (20), similar to over-fed babies born SGA (92). Several studies summarized above (25, 26, 28, 60) highlighted the signature finding among children with PSS: CUG upon removal from the deleterious environment into a more nurturing one, even within the hospital where the children were first evaluated. Not only was rapid growth noted by these investigators and subsequently by many others, but there were also nearly universal salutary changes in the bizarre behaviors that were the benchmarks for considering the diagnoses of PSS. Several of the early descriptive studies also considered the diagnosis of (partial) hypopituitarism (28, 66), but it was Powell et al. (24, 25) who noted hypopituitarism at least with respect to the HPA and GH/IGF-1 axes that was *reversible* upon removal from the toxic environment *without* any specific therapy, whether medical or psychological. These and subsequent studies noted that the GH/IGF-1 system was disturbed most with this syndrome, and that restoration of GH/IGF-1 function could occur quite quickly, even within the first weeks of hospitalization (26, 75, 76).

Many of the data refer to growth as affected by hypopituitarism. That was the state of the knowledge base in the mid-to-late 20^th^ century. There were no studies related to the growth plate in the hierarchy of physiology and pathophysiology of growth in children. In recent years there has been a much greater emphasis and interest in genes within the growth plate, some regulating metabolic factors and cycles that subserve growth. Genome-wide association studies (GWAS), targeted gene sequencing and whole exome or genome sequencing abound (102–104). Although of great interest for primary growth disorders, it is far too early to implicate them in some of the secondary growth disorders, including psychosocial short stature. Perhaps new studies exploring marks in the epigenome will inform how psychosocial stress can affect one’s genome and affect not only that individual, but perhaps in the next generation.

The blending of the biological (nutritional, neurological, and endocrine) and behavioral/cultural/intellectual spheres defines psychoneuroendocrinology.

### Summary

The various conditions described in this review are summarized in **Table 15**.

**Table 15:** Summary of the major similarities and differences among the various conditions noted in this review: hospitalism, feeding studies for malnourished children after World Wars I and II, malnourished children in general, Maternal Deprivation Syndrome (PSS, type 1), psychosocial short stature with hypout hyperphagia (PSS IIb).

Type	History	Anthropometry	CUG*	Emotional and behavioral symptoms	Laboratory data	Ref
Hospitalism	Infants in foundling Hospital or for acute or chronic illness	Underweight and slowly growing	NA	Inanition; poorly interactive with care givers	Few but commensurate with starvation	23, 24, 34–37
Feeding studies after WW I and II	Malnourished with slow growth	Height and weight deficits for age	Marked but only those without emotional problems	Mostly those from pre-war homes that were disturbed; or when housed under harsh authority	Hemoglobin increase; more reactive to tuberculin antigen	16–20, 60, 61
Malnourished	Inadequate food intake; infections; diarrhea	Height or length deficit; underweight for height; failure-to-thrive	Slow with proper feeding and care	Relatively uncommon	GH ↑, basal and stimulated; IGF-1 ↓; insulin ↓; with refeeding GH ↓; IGF-1 ↑; insulin ↑; albumin ↑	2, 7, 8, 11–13, 27, 56, 57
Maternal Deprivation syndrome (PSS, Type 1)	Infants from disturbed homes; maternal-child dyad important	Length and weight deficits for age	usually when properly fed and psychosocial aid to family	Poor interaction with caregiver; often the mother	Consistent with malnourishment; GH levels usually measurable (basal)	9, 10, 49, 50
PSS IIa	Children >2 years Bizarre behaviors including gorging and vomiting (hyperphagia); sleep disturbance and night wandering often in search of food; pain agnosia; abnormal and disturbed relationship with primary care-giver; temper tantrums; poor peer relationships	Slow growth often with appropriate weight for height	Marked with removal from the disturbed environment even without hormonal or behavioral therapy	Children >2 years Bizarre behaviors including gorging and vomiting (hyperphagia); sleep disturbance and night wandering often in search of food; pain agnosia; abnormal and disturbed relationship with primary care-giver; temper tantrums; poor peer relationships	Must be tested early after removal from home; GH deficient whether physiologic or after stimuli to GH secretion; IGF-1 ↓; marked reversal quickly on removal from the disturbed environment; ACTH deficit is variable	25, 26, 42–47, 51–55, 70, 72–88
PSS IIb	Children >2 years; Many of the same bizarre behaviors, but without hyperphagia	Slow growth often with appropriate weight for height	Perhaps less marked with removal from the disturbed environment	Many of the same bizarre behaviors, but without hyperphagia	Without GH/IGF-1 deficit	51–55

*CUG is catch up growth.

## Author’s Note

AR conceived the topic from many decades of communications with the late Robert M. Blizzard, who oversaw the first studies to show reversible hypopituitarism as the fundamental mechanism for growth faltering in children with psychosocial short stature. We had dozens of conversations about this condition and its various presentations. I dedicate this report to him.

## Author Contributions

The author confirms being the sole contributor of this work and has approved it for publication.

## Conflict of Interest

The author declares that the research was conducted in the absence of any commercial or financial relationships that could be construed as a potential conflict of interest.
